# Microemulsion formulation of a new biopesticide to control the diamondback moth (Lepidoptera: Plutellidae)

**DOI:** 10.1038/s41598-018-28626-0

**Published:** 2018-07-12

**Authors:** Hainan Shao, Na Xi, Yalin Zhang

**Affiliations:** Key Laboratory of Plant Protection Resources and Pest Management, Ministry of Education, College of Plant Protection, Northwest A&F University, Yangling, Shaanxi 712100 China

## Abstract

This study was designed to develop a microemulsion formulation of norcantharidin for the control of the diamondback moth (DBM), *Plutella xylostella* (Linnaeus), a notorious pest of brassica crops worldwide. The oil phase was screened and selected based on norcantharidin solubility while the surfactants were selected on the basis of their efficiency to form microemulsion. Optimized batches were selected using pseudo ternary phase diagrams. The microemulsion system were stabilized using mixtures composed of norcantharidin, surfactants (Tx13 and Tw80), and cosurfactant (ethanol). Its physicochemical characteristics were also demonstrated to have a higher cloud point than 72 °C as well as good thermodynamic and dilution stability. In additon, a subsequent insecticidal bioassay indicated that the acute LC_50_ for norcantharidin microemulsion to *P*. *xylostella* was estimated to be 12.477 mg/L (11.58–13.41, 95% CL). Our results provide an environment-friendly promising alternative to control *P*. *xylostella* and possibly contribute to ameliorating any pesticide resistance in *P*. *xylostella*.

## Introduction

Pesticide formulations play important roles in delivering agrochemicals to target sites and increasing their efficacy. Conventional formulations (e.g., emulsifiable concentrate) based on petroleum and organic solvents are gradually being replaced by water-based pesticide formulations (such as microemulsions and nanoemulsions) which have drawn wide attention during recent years^1^. In particular, microemulsion has been extensively used as pesticide vehicles because of their unique advantages^2–5^. A microemulsion is a single optically isotropic and thermodynamically stable liquid solution, having an ultramicroscopic droplet size (typically below 100 nm) and being completely water-dilutable^6,7^. Generally, microemulsions are optically transparent or translucent dispersions of pesticides in water presenting solubilization of the surfactants and/or co-surfactants^7,8^. This small droplet size and lowered surface tension of the whole system may produce higher pesticide efficiency and ease any tendency to coalesce. Oil-in-water (O/W) and water in oil (W/O) are two basic types of microemulsions, within which the O/W microemulsion can improve the solubility of poorly water-soluble active ingredients. Some studies have shown that microemulsions are superior to traditional formulations and exhibit superior control efficiency when compared to traditional commercial pesticides^3,7,9^. Meanwhile, it has been reported that λ-cyhalothrin and triadimefon microemulsions possess better thermodynamic stability, properties and efficacy than their other formulations^10,11^.

The diamondback moth (Lepidoptera: Plutellidae) causes devastating losses of cruciferous vegetables such as cabbage, broccoli and cauliflower, and also damages a few other field crops^12–14^. Application of synthetic insecticides has become more prevalent as a control strategy than other methods. However, resistance and cross-resistance problems in *Plutella xylostella* are increasing whenever a chemical pesticide or mixture of pesticides are used intensively for any extended period because there are few classes of available insecticides with limited mode actions^15^. *P*. *xylostella* has developed resistance to over 94 active ingredients, including the newer insecticides spinosad and emamectin benzoate, based on a research by Michigan State University^4^. Insectile resistance and cross-resistance problems are increasing and the discovery of new products meeting the increasing standard of environmental and toxicological safety is urgent. But development of new synthetic pesticides becomes increasingly more expensive and difficult. In order to decrease insect resistivity,operational programmes may apply new biopesticides and other classes of insecticides in sequence, rotation or mosaics, using of compounds acting on different target sites to avoid selecting for any specific type of resistance.

Norcantharidin is a demethylated analogue of cantharidin; showing the same mode of action, norcantharidin is an inhibitor to protein serine/threonine phosphatases *in vivo*^16^. Protein serine/threonine phosphatases play important roles participating in a series of metabolic activities and their domain alignments show a high similarity in eukaryotic cells^17^. Chen *et al*. (2014) observed that norcantharidin strongly inhibited five protein serine/threonine phosphatase genes of *P*. *xylostella in vitro*^18^. Norcantharidin has also been utilized as a pesticide in pest control and against pathogenic fungi^19,20^. Norcantharidin therefore has potential to be an eco-friendly pesticide in the case of the control the diamondback moth for ecologically sustainable development because of its fast degradation rate and new mode of action^21^.

Lower compound loading capacity, high cost and various side effects are the significant shortcomings of currently available formulations. To avoid these shortcoming, the development of new pharmaceutical formulations of norcantharidin for agricultural administration is desirable. Given the above drawbacks, the main focus of this study is to establish a pharmaceutical microemulsion for the effective application of norcantharidin as a potentially effective and safe pesticide to control *P*. *xylostella* where cyclohexanone, ethyl butyrate and dimethyl formamide are used as solvents. The impact of emulsifiers (Tx13 and Tw80) and a cosurfactant (ethanol) on phase behavior was systematically studied by pseudo-ternary phase diagrams. Characterizations of the microemulsion–such as conductivity, dilution stability, cloud point and other physiochemical parameters–were selected to evaluate the optimal norcantharidin microemulsion system. Finally, insecticidal activity of the norcantharidin microemulsion was analyzed against 3rd instars of *P*. *xylostella* in a leaf-dip bioassay (48 h). In addition, norcantharidin microemulsion could contribute to decreasing the resistance of *P*. *xylostella* to mostly synthetic insecticides though mixing with other classes of insecticides, or by application in sequence or rotation in agricultural practice.

## Results and Discussion

### Component selection of microemulsions

The selection of appropriate solvents plays a key role in preparing microemulsions, as only the dissolved active compounds can be distributed on the leaves. Active ingredient’s solubility in the oil phase has a significant impact on the capability of the microemulsion to keep the compound in dissolved condition. In addition, an oil having low ingredient solubility would require a higher amount of oil to incorporate the desired dose of an active ingredient. In order to keep the miscibility of oils, higher amont of surfactants and cosurfactants would be needed which might increase both the side effects and the toxicity of the system^22^. Our results showed that norcantharidin attained a greater solubility of 50 g/L when dimethylformamide, cyclohexanone and ethyl butyrate were mixed in a ratio of 1 : 1 : 2 (v/v/v). Therefore, the content of norcantharidin was maintained at 50 g/L for the subsequent studies. The choice of surfactant is critical for the formulation of microemulsions, as it helps reduce the interfacial tension by forming a film at the oil-water interface resulting in the spontaneous formation of microemulsions^23^. It was reported that mixed surfactants decrease the minimum weight fraction of surfactant required to solubilize equal weights of water and oil into a single phase^24^. Meanwhile, Fanun reported that the mixture (blend) of surfactants improves the surfactant’s partitioning at the water-oil interface by increaing the solubility of mixed surfactants in both oil and water thus increasing the stability of the amphiphilic film^25,26^. Therefore, mixed surfactants were used in our study. As shown in Table 1, Tx13 and Tw80 were the most appropriate surfactants due to forming a single continuous phase with a transparent appearance. Various Tx13-to-Tw80 weight ratios (1 : 1, 1 : 2, 1 : 3, 2: 1, 2 : 3, 3 : 1 and 3 : 2) were chosen to determine the optimum norcantharidin formulation. The best ratio (w/w) of the surfactant Tx13 to Tw80 was 1 : 1 providing good thermodynamic stability (Table 2). Non-ionic surfactants are often used for the screening of surfactants because they are less influenced by ionic strength and changes in solution pH and generally are considered as biocompatible and safe to non-target organisms^22^.

**Table 1:** Assay of screening optimal surfactants.

Surfactants	Appearance	Appearance after 24 h
Agricultural emulsifier 500	White, turbid	Destabilization, crystallization
Agricultural emulsifier 0209	Semitransparent solution	Slight destabilization
Agricultural emulsifier 0123	Semitransparent solution	Slight destabilization
Agricultural emulsifier 400	White, emulsion	Evident destabilization
Agricultural emulsifier 790	White, emulsion	Evident destabilization
Agricultural emulsifier 1601	Transparent solution	Transparent
Agricultural emulsifier 408	White, turbid	Destabilization, crystallization
Span-80	White, turbid	Destabilization, crystallization
By-125	Semitransparent solution	Slight destabilization
Tw80	Transparent solution	Transparent
Np-4	Semitransparent solution	Slight destabilization
Emulsifier OP-10	White, turbid	Destabilization, crystallization
Tx13	Transparent solution	Transparent

**Table 2:** Results of screening the optimal ratio of emulsifiers.

Tw80: Tx13	Appearance	Appearance after 24 h	Low temperature stability	High storage stability	Cloud point (°C)
1 : 1	Transparent	Transparent	Transparent	Transparent	>72
1 : 2	Transparent	Transparent	Transparent	Transparent	>54
1 : 3	Transparent	Transparent	Transparent	Transparent	>54
3 : 1	Turbid	—	—	—	—
3 : 2	Turbid	—	—	—	—
2 : 1	Transparent	Transparent	Some precipitation	—	—
2 : 3	Transparent	Turbid	—	—	—

“–”: not tested.

### Construction of pseudo-ternary phase diagrams

**Figure 1:**
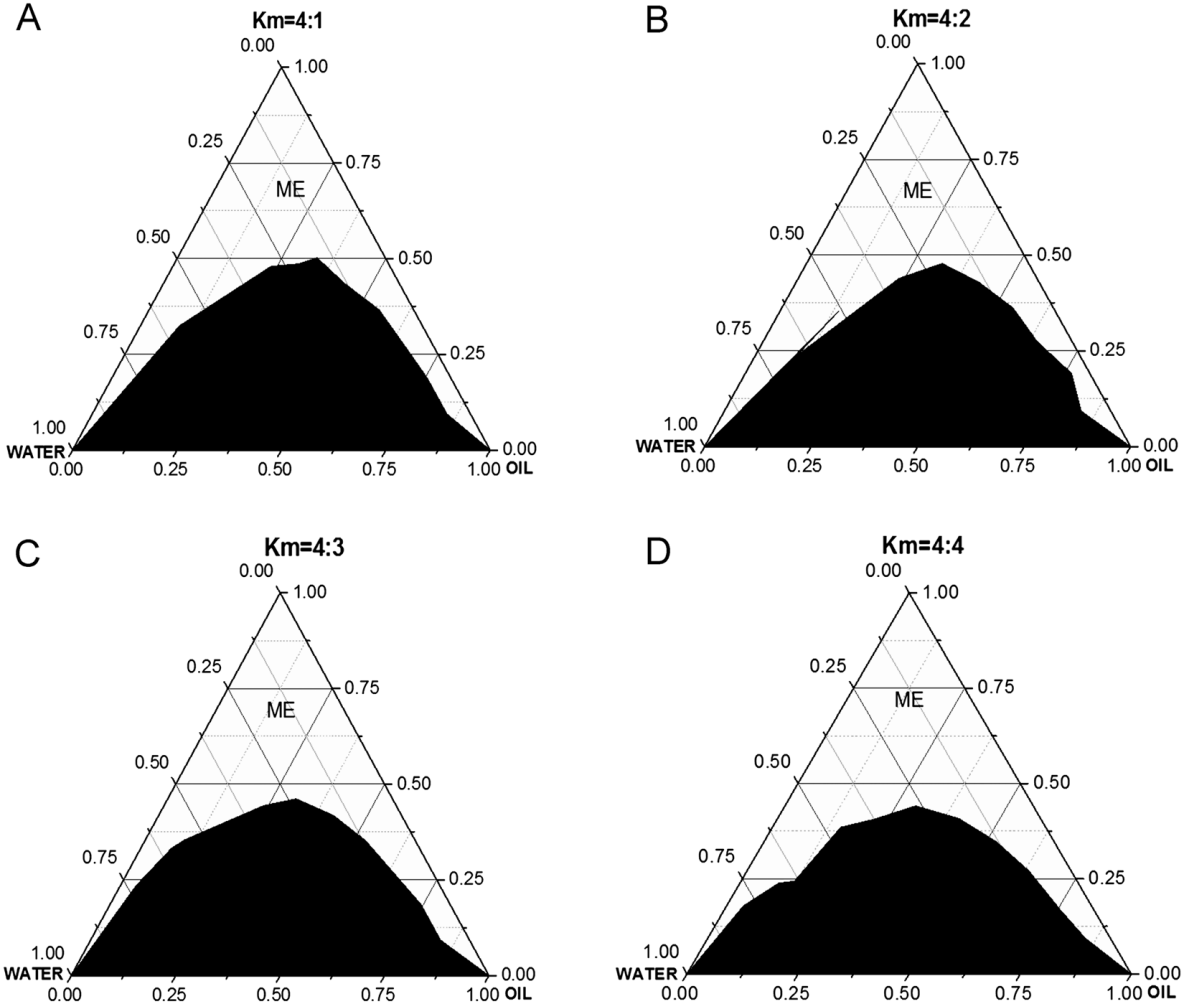
The pseudo-ternary phase diagrams of oil-surfactant-H_2_O system at different ratios of emulsifiers-to-cosurfactant, (**A**) Km = 4 : 1, (**B**) Km = 4 : 2, (**C**) Km = 4 : 3, (**D**) Km = 4 : 4.

In this study, ethanol was used as the cosurfactant. As previously reported, the addition of proper mass short-chain alcohols into non-ionic o/w as a cosurfactants helps in decreasing interfacial free energy and tension and balancing hydrophilic and hydrophobic values of the system by insertion into the interfacial layer^27^. To seek the optimal proportion of surfactants and cosurfactants in ME, the pseudo-ternary phase diagrams were constructed by the water titration method^23,28^. The effects of different surfactants-to-cosurfactant ratios (Km = 4 : 1, 4 : 2, 4 : 3 and 4 : 4) on the phase behavior of the water/Tw80-Tx13/ethanol/norcantharidin system were investigated. The Tw80-Tx13 ratio was kept constant at 1 : 1. The translucent microemulsion regions were presented in the phase diagrams without a distinct phase inversion from w/o to o/w microemulsion^29^. As shown in Fig. 1, the microemulsion regions change with the addition of different ratios of cosurfactant. For the mono-phase region, the smallest (32.75%) was formed when Km was 4 : 1 (Fig. 1A), whereas the largest (39.47%) was detected when Km reached 4 : 4 (Fig. 1D), suggesting that 4 : 4 was the most appropriate Km to form a stable norcantharidin microemulsion. Their characteristics were assessed and the optimum formulation was determined to be a 5% norcantharidin microemulsion system with 12% (w/w) dimethyl formamide, cyclohexanone and ethyl butyrate (1 : 1 : 2), 15% (w/w) Tw80 and Tx13 (1 : 1), 15% (w/w) ethanol, and 58% (w/w) water (Table 3).

**Table 3:** Results of norcantharidin microemulsion screening assay.

Oil/SAA/H_2_O	Appearance	Freeze thaw cycles	Low temperature stability	High storage stability	Cloud point (°C)
12/26/62	Transparent, Tyndall effect	Froze	Transparent	Transparent	>72
12/30/58	Transparent, Tyndall effect	Transparent, no precipitate	Transparent	Transparent	>72
15/30/55	Transparent, Tyndall effect	Slightly turbid	Turbid	Transparent	>72
15/36/49	Transparent, Tyndall effect	Turbidity and crystallization	Turbid	Transparent	>72

### Conductometry and structure of norcantharidin microemulsion

**Figure 2:**
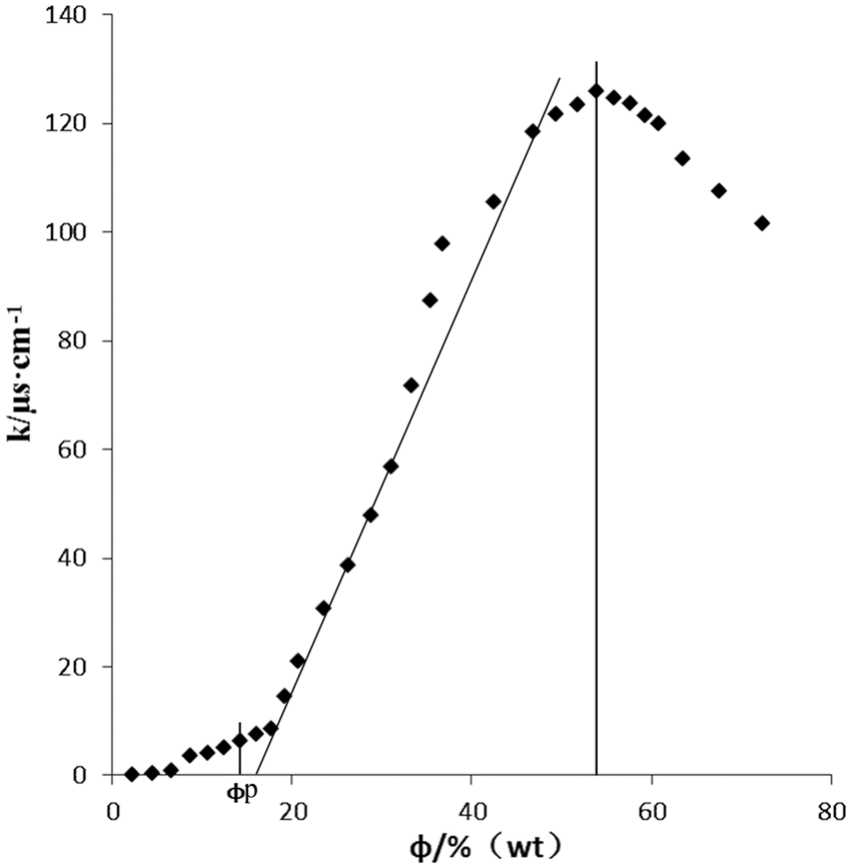
Graph of the relationship of the electrical conductivity with the water amount in the mixed SAA/mixed oil/water system (standard water, 342 mg/L) SAA: the ratio of emulsifiers-to-cosurfactant (4 : 4).

Conductivity is a key technique to detect the percolation threshold in microemulsions^30^. With the fixed ratio of mixed oil/surfactants/co-surfactant, the weight ratio of water increases along with linear-type experimental paths by stepwise addition into the mixture. Obviously, four regions exist as indicated in Fig. 2. The electrical conductivity σ of the selected oily mixture was almost zero and only increased slightly when the volume fraction of water was low. When a water volume fraction (*φ*_w_) reached a critical point (*φ*_p_), termed the percolation threshod, a dramatic change in conductivity is recorded with any further increase in water content; this is when the structure of the microemulsions has converted to a biocontinuous type or reached the emulsification boundary^30,31^. We ascribe the linear increase in σ to the formation of water cylinders or channels in an oil phase due to the attractive interaction between the spherical micro droplets of the water phase in the w/o microemulsion^27^. Accordingly, this steady increase the conductance is attributed to the larger water channels and particle size. When σ reaches the maximum value (1.25 S m^−1^, *φ*_w_ ≈ 40%), then a phase inversion from reverse swollen micelles (w/o) to direct micelles (o/w) is suggested^23,32^. Thereafter, σ values then drop because of dilutional impacts^33^. The structure of the microemulsion from w/o to a discontinuous phase and then o/w using titration is indicated by the derivative of the conductivity (Fig. 2).

### Stability of microemulsions

#### Low and high temperature storage stability

After storage at 0 and 54 °C for 14 days, the appearance of the prepared norcantharidin microemulsion remained transparent and uniform, without any precipitate or phase separation. The decomposition rates of norcantharidin was 2.13% and 2.51%, which means there is no significant change in the stability of the microemulsion.

#### Freeze-thaw cycles

For this obtained norcantharidin microemulsion, thermodynamic stress tests were conducted using freeze-thaw cycles between −21 °C and 25 °C. At each temperature, the samples were stored for no less than 48 h. No freezing or floating oil was detected in the tested samples.

#### Cloud point

Cloud point is one of the specific characteristics of microemulsions. As the temperature increases, the solubility of the non-surfactant decreases, resulting in a turbid mixture. The cloud point occurred at a temperature greater than 72 °C. After centrifuge tests, there was no precipitation or phase separation of norcantharidin microemulsion, suggesting a good physical stability.

#### Dilution stability

**Figure 3:**
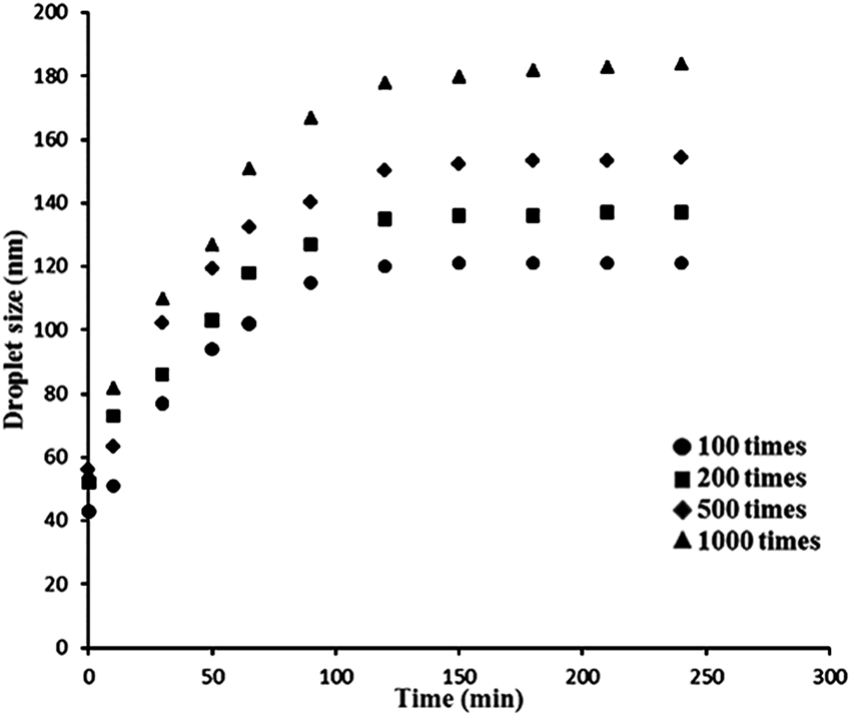
The variation of droplet size of 5% norcantharidin microemulsion based on time under different dilution times with standard water (342 mg/L).

To ensure the homogenous distribution of a chemical during field application for pest management, agrochemical microemulsion formulations are required to retain their integrity against dilution with water^34^. DSL analysis was conducted to assess the homogeneity of the microemulsion sample. DSL analysis provided evidence for the presence of transparent microemulsion systems, with a narrow size distribution for all samples. The microemulsions remained homogeneous and transparent during the dilution process, although DSL results indicated an increase in the droplet size along with the augmented dilution ratio (Fig. 3). The increase of droplet radii against dilution may be attributed to a decrease in volume fraction or to an attractive influence between the diluted microemulsion droplets. In diluted microemulsion systems, the increase in the diffusion coefficient of the droplet may be largely due to the attractive interactions which may ultimaltely result in the increase of the hydrodynamic radii of diluted samples^34^. Similarily, cosurfactant may diffuse from the interface film into the water phase due to the dilution with hard water, leading to the coalescence of the oil phase reducing the increased free energy and interfacial area. Once the diluted microemulsion systems reached a new balance again, the droplet size tended to become stable with time. Therefore, a microemulsion possessing macroscopical dilution stability was obtained^7,35^.

#### Insecticidal activity

Norcantharidin, as an animal-derived variant compound of cantharidin, has shown insecticidal and anti-fungal activities^19,20^. But its agricultural use is restricted to some extent due to poor water solubility. Therefore, norcantharidin microemulsion has been developed in order to improve solubility and improve efficiency. According to Table 4, all 6 commercial insecticides are shown to be highly active against *P*. *xylostella* larvae, among which 5% emamectin benzoate ME had the minimal LC_50_ (0.07 mg/L; 0.06–0.09, 95% CL). Even though cantharidin and norcantharidin showed a higher LC_50_ (6.501 and 12.477 mg/L) to early-stage larvae of *P*. *xylostella* compared to the reminding insecticides, these compounds were used as an insecticide because of their new mode of action, fast degradation and non target effects^21^. Considering the complex and difficult synthesizing procedures^36,37^ as well as scarce origin^38^ of cantharidin, norcantharidin is a good alternative candidate. The 48 h LC_50_ value (95% confidence limits) of the 5% norcantharidin microemulsion for *P*. *xylostella* was found to be 12.477 (11.58–13.41) mg/L.

**Table 4:** Log-dose probit-mortality data for different commercial pesticides tested against 3rd instar of *P*. *xylostella* in a leaf-dip bioassay (48 h).

Insecticides	Formulation	Active ingredients (%)	n^a^	Slope ( ± SE)	Relation coefficient	LC_50_ mg/L (95%CL^b^)	χ^2^
cantharidin		100%	279	2.448 ± 0.55	0.99	17.108 (14.19–21.30)	3.19
norcantharidin		100%	280	3.258 ± 0.82	0.98	49.959 (33.01–63.49)	11.1
cantharidin	EC	1%	278	2.341 ± 0.35	0.95	6.501 (5.30–8.00)	3.15
norcantharidin	ME	5%	280	6.124 ± 1.00	0.97	12.477 (11.58–13.41)	0.97
azadirachtin	ME	1%	276	4.032 ± 0.30	0.94	0.35 (0.31–0.39)	1.19
emamectin benzoate	ME	5%	280	2.805 ± 0.47	0.98	0.07 (0.0.06–0.09)	6.46
Spinosad	SC	60 g/L	276	0.956 ± 0.83	0.93	0.73 (0.67–0.81)	6.54
Indoxacarb	SC	30%	280	0.911 ± 0.12	0.92	0.933 (0.86–0.95)	8.16

^a^Number of larvae tested, ^b^Confidence interval;

EC: emulsifiable concentrate, ME: microemulsion, SC: suspension concentrate.

Both cantharidin EC and norcantharidin ME exhibited better activity against *P*. *xylostella* than did their pure products (on average, 163.16% and 300.41% higher as shown in LC_50_, respectively) (Table 4). Previous studies also demonstrated that commerical formulations often exhibited higher toxicity to the target pest than did the corresponding active ingredients^39,40^. One possbile reason is that marketed pesticides are composed of active ingredients and quite low amount of emulsifiers and/or short chain alcohols which would increase the bioavailability of the pesticide by increasing the solubility or compatibility of the active ingredients and also facilitate adsorption, penetration and translocation of the active ingredients into the targets^41^. Lei *et al*. (2012, 2014) and Wang *et al*. (2017) found that microemulsions showed a significantly higher lehtal activity than other types of formulations^3,7,9^. Another possible explanation is that as opposed to a single solvent, traditional chemical products are more effective when mixed solvents are employed^3^. Therefore, norcantharidin microemulsion showed a much better activity than a correspondingly pure active ingredient alone (Table 4).

When tested at the recommended rates, all six pesticides caused high mortality of third-instar larvae of *P*. *xylostella* (Table 5), especially spinosad and emamectin benzoate which showed the highest toxicities, with a larvae death rate up to 100% within 24 h. In contrast, the remaining pesticides caused over 60% mortality of the 3rd instars within 24 h and the larvae completely died during the next 24 h. These results are in accordance with Liu *et al*. (2003) and Zhao *et al*. (2006), who reported that emamectin benzoate and spinosad had high level of intrinsic toxicity to early-stage larvae of *P*. *xylostella*^42,43^.

**Table 5:** Efficacy and larvicidal activities of different pesticides against susceptible *P*. *xylostella* at commercially recommended rate.

Insecticides	Concentration (mg/L)	Larval mortality under different times after treatment (%) (±SD)		
		12 h	24 h	48 h
Control		0.0	0.0	0.0
Cantharidin	15	34.5 ± 1.48^b^	82.3 ± 0.78^b^	100^a^
Norcantharidin	30	21.3 ± 0.83^c^	56.8 ± 0.45^c^	95.6 ± 3.24^a^
Azadirachtin	10.5	35.9 ± 1.02^b^	56.4 ± 1.26^c^	100^a^
Emamectin benzoate	5	90.0 ± 0.51^a^	95.0 ± 3.14^a^	100^a^
Indoxacarb	45	12.5 ± 0.12^d^	52.5 ± 5.47^c^	97.5 ± 3.15^a^
Spinosad	30	92.5 ± 0.05^a^	100^a^	100^a^

Data in the same column followed by a different letter are significant (*P* < 0.05).

Over the past decades, world wide and long-term use of synthetic pesticides has led to insect resurgence, pesticide resistance, biomagnifications and tremendous environmental damage although they aim to control pests, pathogen and weeds. The diamondack moth remains a notorious insect pest mainly resulting from its extremely easy to evolution of resistance to many classes of insecticides^44,45^ e. g., spinosad, indoxacrad and emamectin benzoate^42,46,47^. These challenges, coupled with negative effects on the environment and the high costs of the application and use of synthetic pesticides as well as consumers’ increasing demand of safe food are the main impetus to search for safer and biodegradable biopesticides. Biopesticides have attracted tremendous interest because they are eco-friendly^48^. Biopesticides and their derivatives are considered to be good alternatives to synthetic pesticides in controlling this harmful insect and have been successfully used in agriculture^49–52^.

In this study, we successfully developed an effective and novel norcantharidin microemulsion system with optimal characteristics, which showed a good control effect on the diamondback moth in the laboratory. Microemulsions can help in improving the bioavailability of poorly solube chemicals and ameliorating the adverse impacts of petroleum and organic solvent based-pesticides on the environment by way of altering their formulations. Further studies should be conducted to estimate the efficacy of practical application of norcantharidin microemulsions in the field and in combination with other insecticides to heighten pesticides’ effectiveness, reduce chemical consumption and lower agrochemical toxicity. Biopesticides are components of Integrated Pest Management (IPM) and contribute to delaying the development of insect resistance.

## Materials and Methods

### Insects and pesticides

Insecticide-susceptible stains of *P*. *xylostella* were reared under optimal growth conditions (25 ± 2 °C, 50% relative humidity and a photoperiod of 16 L : 8 D) without insecticide selection. Norcantharidin (>98%) was purchased from Alfa Aesar Chemical Co. Ltd (Haverhill, MA, USA). The emulsifiers Tx13 (alkylphenol polyoxyethylene) and Tw80 (polyoxyethylene sorbitan monooleate) (Hai’ an Petrochemicals, Jiangsu, China) were of commercial grade. Formulated insecticides used for bioassays included 1% azadirachtin microemulsion (ME) (Kepu Biochemical Co., Ltd), 5% emamectin benzoate microemulsion (ME) (Yinnong Technologies Co., Ltd), 60 g/L spinosad suspension concentrate (SC) (Dow AgroSciences LLC), 30% indoxacarb suspension concentrate (SC) (Jiangsu Jianpai Agrochemical Co., Ltd), 1% cantharidin emulsifiable concentrate (EC) (homemade) and 5% norcantharidin microemulsion (ME) (homemade).

### Solvent screening

The solubility of norcantharidin in various solvents were determined to find out the appropriate solvents with a good capacity for norcantharidin in ME. The solubility of norcantharidin in mixed solvents was also measured. 0.5 g norcantharidin was added into 10 ml of each solvent at various temperatures, and the resultant mixtures were shaken reciprocally for 72 h followed by centrifugation for 30 min at 10000 rpm. The solvent (s) which can completely dissolve norcantharidin and the appearance always maintained transparent was chose for further experiments.

### Surfactant screening

In order to screen and select the most effective surfactant (s), mixed solutions of equal masses of active ingredient (norcantharidin) and various surfactants were incubated using a thermobath at 20 °C for 24 h. The appearance of each mixture, the temperature range over which transparency was maintained, and low temperature and freezing stability were used to determine the quality of the obtained microemulsion formulation.

### Pseudo-ternary phase diagram construction

To ascertain the concentration ranges of compositions in microemulsion range, pseudoternary phase diagrams were displayed using a water titration method at ambient temperature (25 ± 1 °C). The solution in which norcantharidin completely dissolved was the oil phase. At the desired Km (4 : 1, 4 : 2, 4 : 3 and 4 : 4), the mixture of surfactants (Tw80 and Tx13) and cosurfactant (ethanol) was set as an independent variable in the phase diagram. Then the weight ratios of the oil phase and the mixture (surfactant and co-surfactant) were varied from 1 : 9 to 9 : 1. Water was added to the oily mixture dropwise with gentle stirring. The appearance of the whole system underwent a change from transparent to turbid or the reverse, and a corresponding phase boundary was obtained.

### Conductivity

The conductivity change of the microemulsion system with the increasing ratio of water was analyzed by a low frequency conductometer (DDS-307A, Shanghai Precision Scientific Instrument Co., Shanghai, China) at 25 ± 0.1 °C. The conductivity change has been typically used as an indicator to determine the phase inversion of the microemulsion from O/W to W/O.

### Characterization of ME

#### Droplet size measurement

The average droplet size of the norcantharidin microemulsion and its changes against different multiples of dilution (100, 200, 500 and 1000 times) with standard hard water (content of Ca^2+^, Mg^2+^ were 342 mg/L) were measured by dynamic light scattering (DLS) using a Zetasizer Nano-ZS (Malvern Instruments). The backscatter measurement was performed at a fixed angle of 173° at 25 °C. The droplet size of each dilution was measured with four repetitions.

#### Centrifugation

The centrifugation of formulations at 13,000 rmp for 30 min and at 4000 rmp for 4 h was carried out to assess the physical stability of ME.

#### Thermodynamic stability

The microemulsion prepared according to the above methods was kept in place at 0 and 54 °C for 14 days; then the appearance, mobility, emulsifying properties and the extent of active material were measured. Meanwhile, samples were stored at −18 °C for 7 days to observe the stability of the microemulsion. Finally, the cloud point was measured by gently stirring the sample in a water bath, with the temperature set to increase at 2.0 °C/min intervals, until the microemulsion became observably turbid^53^.

#### Content of active ingredients in the microemulsion

The concentrations of norcantharidin were determined by GC analysis using an Agilent 4890D (Santa Clare, CA, USA) with a HP-5 capillary column (15 m × 0.53 mm × 1.5 μm) filled with 101 silylations supporter. The injector and detector temperature were 230 °C and 260 °C respectively. Nitrogen was the carrier gas at a constant flow-rate (30 ml/min), hydrogen at 2.0 kg/cm^2^ and air at 2.55 kg/cm^2^. The initial oven temperature was 150 °C for 3 min; then the temperature was raised at a rate of 10 °C/min to 260 °C. A standard concentration of norcantharidin was used to construct a calibration curve. Each microemulsion sample was repeated 4 times, with the concentration of norcantharidin was calculated according to the following formula:

$${\rm{Concentration}}( \% )=\frac{{{\bf{R}}}_{2}\times {{\bf{M}}}_{1}\times {\bf{100}}}{{{\bf{R}}}_{1}\times {{\bf{M}}}_{2}}$$

Where: **R**_**1**_: ratio of the area of norcantharidin to the internal reference in the standard solution; **R**_**2**_: ratio of the area of norcantharidin to the internal reference in the sample solution; **M**_**1**_: the weight of standard norcantharidin (g); **M**_**2**_: the weight of the tested microemulsion sample (g).

#### Bioassays

Commercial pesticides (norcantharidin microemulsion, cantharidin emulsifiable concentrate, indoxacarb suspension concentrate, spinosad suspension concentrate, emamectin benzoate microemulsion and azadirachtin microemulsion) and active ingredients (norcantharidin and cantharidin) were prepared as serial dilutions with distilled water containing 0.1% TritonX-100; the solution containing 0.1% TritonX-100 only was taken as a control. Leaf discs (Ø 1.0 cm) of cabbage (*Brassica oleracea*) were dipped into prepared solutions for about 10 s and dried for about 1 h at ambient temperature. Ten dipped leaf discs and ten third-instar larvae were placed into a sterilized plastic petri dish (Ø 9.0 cm) containing a humid filter paper to maintain freshness. Four petri dishes with a total of 40 *P*. *xylostella* larvae of each sample were incubated in a growth chamber (25 ± 1 °C, 65% RH with a photoperiod of 16 : 8 (L: D)) and mortality of *P*. *xylostella* was determined after 24 and 48 h. Larvae that did move when gently touched with a brush were considered dead. Where the mortality of controls exceeded 10%, the experiments were repeated.

#### Statistical analysis

The pseudo-ternary phase diagram was constructed using Origin 9.1. Where necessary, bioassay data were corrected for the control mortality^54^. Values presented here represent a mean of four repetitions; concentration-response data were calculated by probit analysis. The other statistical analyses were conducted using SPSS 21.0. The mortality of different pesticide-treated samples were compared using a one-way ANOVA. Values were considered to be significantly different at *P* $<$ 0.05.
